# Multifunctionality and Diversity in Bacterial Biofilms

**DOI:** 10.1371/journal.pone.0023225

**Published:** 2011-08-05

**Authors:** Hannes Peter, Irene Ylla, Cristian Gudasz, Anna M. Romaní, Sergi Sabater, Lars J. Tranvik

**Affiliations:** 1 Limnology, Department of Ecology and Genetics, Uppsala University, Uppsala, Sweden; 2 Department of Environmental Sciences, Institute of Aquatic Ecology, University of Girona, Girona, Spain; 3 Catalan Institute for Water Research (ICRA), Girona, Spain; Universidad Miguel Hernandez, Spain

## Abstract

Bacteria are highly diverse and drive a bulk of ecosystem processes. Analysis of relationships between diversity and single specific ecosystem processes neglects the possibility that different species perform multiple functions at the same time. The degradation of dissolved organic carbon (DOC) followed by respiration is a key bacterial function that is modulated by the availability of DOC and the capability to produce extracellular enzymes. In freshwater ecosystems, biofilms are metabolic hotspots and major sites of DOC degradation. We manipulated the diversity of biofilm forming communities which were fed with DOC differing in availability. We characterized community composition using molecular fingerprinting (T-RFLP) and measured functioning as oxygen consumption rates, the conversion of DOC in the medium, bacterial abundance and the activities of five specific enzymes. Based on assays of the extracellular enzyme activity, we calculated how the likelihood of sustaining multiple functions was affected by reduced diversity. Carbon source and biofilm age were strong drivers of community functioning, and we demonstrate how the likelihood of sustaining multifunctionality decreases with decreasing diversity.

## Introduction

In the face of a global decline in biodiversity, several recent reviews and meta-analyses reported a positive, but saturating relationship between biodiversity and individual ecosystem functions [1], [2], like biomass accrual [3], resource utilization [4] and temporal stability [5]. Saturation of functioning at high levels of diversity suggests the presence of functionally redundant species. Microbes encompass the highest diversity of all life forms on Earth [6], [7] and they drive a bulk of ecosystem functions. However, the roles microbial diversity play in controlling ecosystem functioning remain largely overlooked [8]. Microbial diversity is commonly neglected in biogeochemical models, which implicitly assume that large-scale ecosystem functions are independent from microbial diversity [9].

While early studies about biodiversity and ecosystem functioning focused mainly on single ecosystem processes [8], [10], Hector and Bagchi [11] addressed the importance of simultaneous effects of biodiversity on multiple ecosystem functions. They found that the number of species required to sustain ecosystem functioning increased with the number of processes considered. Gamfeldt and coauthors [12] show that analyzing a single response variable overestimates the amount of functional redundancy. Functional redundancy for a single ecosystem function occurs if several species perform the same function. When multiple functions are performed at the same time, complementarity of species across functions is expected to reduce multifunctional redundancy [12]. This concept requires trade-offs for species to perform different functions [8]. Such trade-offs could arise because some functions conflict with other functions (e.g. e.g. productivity and stress tolerance [13]). Therefore, a lesser degree of redundancy is expected to emerge when more functions are addressed. Bacterial communities are often assumed to be functionally redundant due to their high abundance, immense diversity, high dispersal capacity, physiological versatility and horizontal gene transfer. However, there are only few empirical tests of the relationship of biodiversity and multifunctionality [13], [14].

In this study, we address the relationships between bacterial diversity and the activities of extracellular enzymes degrading dissolved organic carbon (DOC). DOC is a key component of the carbon cycle of inland waters [15]. The degradation of DOC is mediated primarily by heterotrophic bacteria, which in streams are located predominantly in biofilms [16], [17]. DOC comprises a diverse mixture of different components [18] and its degradation requires a range of hydrolytic and oxidative enzymes [19]. Bacterial extracellular enzymes accumulate in biofilms [20], where they transform high molecular weight compounds to lower molecular weight compounds which are ultimately assimilated by heterotrophic microbes [21]. These enzymatic reactions constitute different ecosystem functions and their interplay is of crucial importance for overall ecosystem functioning.

Here, we manipulated the diversity of heterotrophic bacterial biofilms in laboratory-based bioreactors and measured the activity of a range of extracellular enzymes and the degradation of DOC as ecosystem functions. We hypothesized that individual ecosystem functions are affected by the loss of diversity and we expected to find a strong effect of the manipulation on ecosystem multifunctionality. This was addressed as the likelihood of sustaining multiple extracellular enzymes above a threshold of the maximal activity. We provided two different substrates and measured biofilm composition and function at an early and late stage of biofilm development. We expected a dependency of multifunctionality on substrate availability and that the age of biofilms plays an important role for the establishment of species interactions and the maintenance of multifunctionality.

## Methods

### Bioreactors

A set of 36 bioreactors was installed in a constant temperature room (20°C) and kept dark. The bioreactor consisted of glass columns (app. 200 mL) filled with sintered borosilicate glass beads (90,000∶1 surface to volume ratio) providing a large area for the growth of biofilm [22]. Three-way valves at the in- and outflow of the bioreactors were used as sampling ports and peristaltic pumps supplied the bioreactors with medium at a flow rate of 1 mL min^−1^. Substrate composition was manipulated by providing two types of medium - carbon-free, artificial lake water medium amended with (i) 15 ppm of a mixture of labile and recalcitrant carbon (termed “labile”) or (ii) 15 ppm recalcitrant carbon only (termed “recalcitrant”). The artificial lake water was prepared as described in Bastviken et al. [23] and for the labile treatment amended with a mix of 7.5 ppm of aged stream water DOC and 7.5 ppm of an artificial labile carbon source (3.75 ppm each of cellobiose and the dipeptide leucine-proline, both Sigma-Aldrich, St. Louise, MO, USA). For the recalcitrant treatment, 15 ppm of aged stream water DOC was added to the artificial lake water. The stream water was sampled from a small tributary to Fibyån, Sweden and aged for 2 month at 20°C. It contained 110.8 ppm DOC. Bioreactors were assembled sterilely, and all sampling and handling was conducted under aseptic conditions. The glass beads were muffled at 450°C for 4 h, while the glass reactors, pump tubing (Tygon) and silicone connection tubing were steam autoclaved (121°C for 20 min). During the entire term of the experiment, we monitored bacterial abundance in the outflow as an indicator of community stability in the bioreactors. Retention time for the medium was estimated in a separate reactor by changing from pure water to a saline solution (100 ppt) in the inflow and measuring conductivity in the outflow. Retention time was estimated to be approximately 300 minutes.

### Preparation of the inoculum

A gradient in diversity was established in pre-cultures using a dilution-to-extinction approach [24]. The bacterial community was collected from a stream (Fibyån, Sweden, N 59° 53′ 7″ E 17° 20′ 43″). Sediment and water were sampled, mixed and filtered (GF/F, Whatman, Maidstone, Kent, UK) to eliminate predatory eukaryotes. The number of cells in the inoculum was adjusted to be between 10^7^ and 10^1^ cells using step-wise dilution with sterile stream water. 100 mL batch cultures with particle free (<0.2 µm, Supor, Pall, Lund, Sweden), sterilized stream water (2 times autoclaved at 121°C for 20 min, with a 24h interval) were amended with 5 mL of the inoculum. The cultures grew at 20°C in the dark and bacterial abundance was measured daily for seven days. Thereafter, bioreactors were inoculated at three levels of diversity, using one pre-culture from the largest nominal inoculum size (10^7^ cells) (i.e. high diversity), one culture from a medium nominal inoculum size (10^5^ cells) (i.e. medium diversity) and one culture from the smallest inoculum that consistently supported bacterial growth (10^3^ cells) (i.e. low diversity). At that time, the batch cultures had reached similar abundances, avoiding hidden effects of differences in starting biomass and abundance.

### Acclimation phase

After inoculating the bioreactors, the cells were allowed to colonize the surface of the glass beads for 4 to 7 weeks. We monitored DOC concentration and oxygen consumption (see below for a description of the methods). During the acclimation phase, the medium was recycled, but we replaced approximately half of the medium (4 L) on a weekly basis.

### Tests of ecosystem function

We performed two sets of tests of ecosystem functioning, lasting for 10 days each. The first test was performed after 4 weeks of acclimation (termed “young biofilms”), while the second was performed after 7 weeks of acclimation (termed “old biofilms”). In each test we included the three diversity levels each with six bioreactors, three each of to the labile and recalcitrant carbon treatment. We measured DOC concentration, absorbance at 250 nm, and oxygen in the in- and the outflow of the bioreactors and calculated respiration in each bioreactor by difference. After 10 days, the corresponding bioreactors were opened (destructive sampling) and the glass beads were sampled to measure the activity of five different enzymes (see below). During the colonization period, differences in community composition might have established along the flow path in the bioreactors, hence we sampled the beads along a gradient, with samples from the inflow, the mid section and the outflow of the bioreactors. A subsample of the beads was stored at −80°C for molecular community composition using Terminal Restriction Fragment Length Polymorphism (T-RFLP). Another subsample was covered with 3.7% formaldehyde and stored at 4°C for enumeration of cell abundance on the beads using flow cytometry.

### DNA extraction and T-RFLP

Cells were harvested by sonicating 25 mL of beads in MQ water and filtering the supernatant onto 0.2 µm membrane filters (Supor, Pall, Port Washington, NY, USA), which were stored at −80°C. DNA was extracted using the Ultraclean Soil DNA extraction kit (MoBio Laboratories, Carlsbad, CA, USA). DNA extracts were used as templates for PCR amplification of the 16S rRNA genes (see Text S1). Reactions of the PCR product with the restriction enzymes hae III and hinf I were incubated at 37°C for 16 h [25]. Terminal fragments were sized by electrophoretic separation and detection on a capillary sequencer (ABI 96, Applied Biosystems, Carlsbad, CA, USA). Size and quantity (peak height) of terminal restriction fragments were analyzed using GeneMarker (ver. 1.7) software.

### Bacterial abundance

Bacterial abundance was measured by flow cytometry of Syto13 (Molecular Probes, Invitrogen, Carlsbad, CA, USA) stained cells [26]. Cells in the outflow were sampled daily, fixed with 3.7% final conc. formaldehyde and stored at 4°C. Bacterial abundance on the beads was estimated after short sonication at intermediate power (12 Watts, Microson XL, Misonix, Farmingdale, NY, USA) of beads covered with a 3.7% formaldehyde solution. The samples were analyzed with a Cyflow Space (Partec, Görlitz, Germany) flow cytometer equipped with a 96 well-plate autosampler (see Text S2).

### Dissolved Organic Carbon

Samples for DOC analysis were taken daily, from the in- and outflow of the bioreactors into muffled 17 mL vials. DOC concentration was analyzed immediately using a Sievers 900 TOC analyzer (GE Healthcare, Boulder, CO, USA) with an accuracy range of ±0.5 ppb. At the beginning and the end of the two tests, absorbance at 250 nm was measured using a spectrophotometer (Lambda 40, Perkin Elmer, Waltham, MA, USA) and a 1 cm quartz cuvette. Carbon-specific absorbance at 250 nm (abs250:DOC) was employed to indicate the proportion of humic substances [27].

### Oxygen consumption

Oxygen consumption was measured four times during each test period. Reactor-wide oxygen consumption was estimated as the difference in oxygen concentration between the in- and outflow of each bioreactor normalized for retention time of the medium. A flow-through optode probe (FTC, PreSens, Regensburg, Germany) connected via an optical fiber to a Fibox 3 oxygen meter (PreSens) was used. The measurements were temperature compensated and excess medium was discarded.

### Extracellular enzyme activities

We measured the activity of five extracellular enzymes produced by natural biofilm communities (see Table S1). The activity of β-glucosidase, β-xylosidase, cellobiohydrolase and leucine-aminopeptidase, which are involved in the last steps of the hydrolysis of polymeric compounds (cellulose, hemicellulose, peptides), was determined spectrofluorometrically by using fluorescent-linked artificial substrates (Sigma-Aldrich). The activity of phenoloxidase, which is involved in the oxidation of lignin was measured using L-3,4 dihydroxyphenylalanine (Sigma-Aldrich) following the method of Sinsabaugh et al. [28]. All enzymatic activities were measured under saturating conditions (see Text S3). Fluorescence was measured at 360/465 nm excitation/emission using a plate reader (Ultra 384, Tecan, Switzerland). Blanks were subtracted from the samples to correct for abiotic hydrolysis of the substrate and fluorescent substances in the medium.

### Data analysis

To investigate bacterial community composition, non-metric multidimensional scaling (nMDS) using Bray-Curtis similarities and Principal Component Analysis (PCA) were calculated using PAST (ver. 2.01) [29]. Both analyses are based on relative abundance data obtained by TRFLP. Differences in abundance on the beads, number of OTUs and oxygen consumption rates were analyzed using ANOVA. Variability in the enzymatic activity measurements (ß-glucosidase, ß-xylosidase, cellobiohydrolase, leucine-aminopeptidase, and phenoloxidase) was analyzed by multivariate analyses of variance (MANOVA, SPSS for Windows, ver. 12.0). SIMCA-P+ (ver. 12.0.1.0, Umetrics, Sweden) was used for PLS modeling (projections of latent structures by means of partial least squares) to identify important drivers of ecosystem functioning. PLS is a regression extension of PCA and allows the exploration of relationships between multiple, collinear data matrices of X and Y [30]. The model performance is expressed by R^2^Y (explained variance) and by Q^2^Y (predictive power as estimated by cross validation). Age, carbon source and the diversity treatment were entered as categorical variables while the continuous scores on the first Principal Component axis of the 16S rRNA fingerprint were used as a measure of community composition. Variable influence on projections (VIP) scores are weighted sums of squares of PLS weights across components taking into account the amount of explained variance in Y. Values above 1 (i.e. average influence) indicate important variables, values below 1 are regarded as less important. For model evaluation see Text S4. Variables for MANOVA and PLS were log (*x*+1) transformed to attain homogeneity of variance and normal distribution.

In order to address multifunctional effects of the diversity treatment we followed the logics outlined in Gamfeldt et al. [12] and defined a specific level of enzyme activity sufficient to sustain community functioning. If any of the individual activities dropped below this threshold, we would consider this specific function lost and consequently the likelihood of sustaining multifunctionality would be impaired. We started with a threshold of 50% of the maximal enzyme activities for each enzyme. This means that as long as the community is able to perform 50% of the maximal enzyme activity in all samples, we would regard the function retained. If functioning for a specific enzyme dropped below 50%, this was considered as a loss of function. We calculated the probability that the activities of all five enzymes surpassed the threshold, in at least one of the three positions sampled along the flow path in the bioreactor. Hence, a value of 1 indicates that all five extracellular enzymes are performed above the threshold, while a value of 0 indicates that none of the enzyme activities reached above the threshold level. Moreover, we investigated the effect of increasing the threshold to 0.75 and 0.9 of the maximum activity.

## Results

### Community Composition

**Figure 1:**
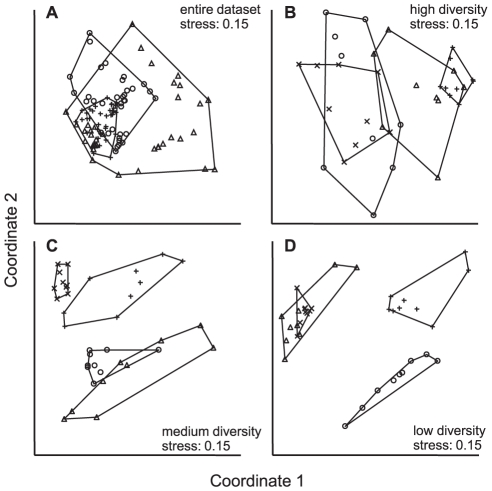
Non-metric multidimensional scalings (nMDS) of the 16S rRNA genes community profiles. Panel (A) shows the entire dataset. Panel (B) shows the high diversity treatment, panel (C) the medium and panel (D) the low diversity treatments. In panel (A), outlines indicated the diversity treatments: + high diversity, ○ medium diversity and Δ low diversity. In panel B, C and D the outlines indicate the treatments: ○ labile/young, + labile/old, x recalcitrant/young and Δ recalcitrant/old.

In total, we detected 77 different operational taxonomic units (OTUs) by T-RFLP, with 34 OTUs detected by the restriction enzyme hinf I and 43 by hae III. On average, 14±2 different OTUs per sample were detected, with no difference between the diversity treatments (ANOVA, _MS 10.2, d.f. 107_ P = 0.12). Hence, a similar number of dominant taxa established in the bioreactors at all levels of diversity. However, the high diversity treatment clustered closely together in the nMDS analysis, while the medium and low diversity samples occupied more space in the bivariate plane (Fig. 1A). Separate analysis of the high, medium or low diversity treatments revealed the impact of age of the biofilms and of carbon source composition, while differences in community composition from the in- to the outflow were not obvious. Under high diversity, the outlines of the treatments overlap considerably, although they appear clearly separated by age (Fig. 1B). The separation of communities by carbon source composition and age became more evident when diversity was reduced (Fig. 1C and 1D).

### Functioning

**Figure 2:**
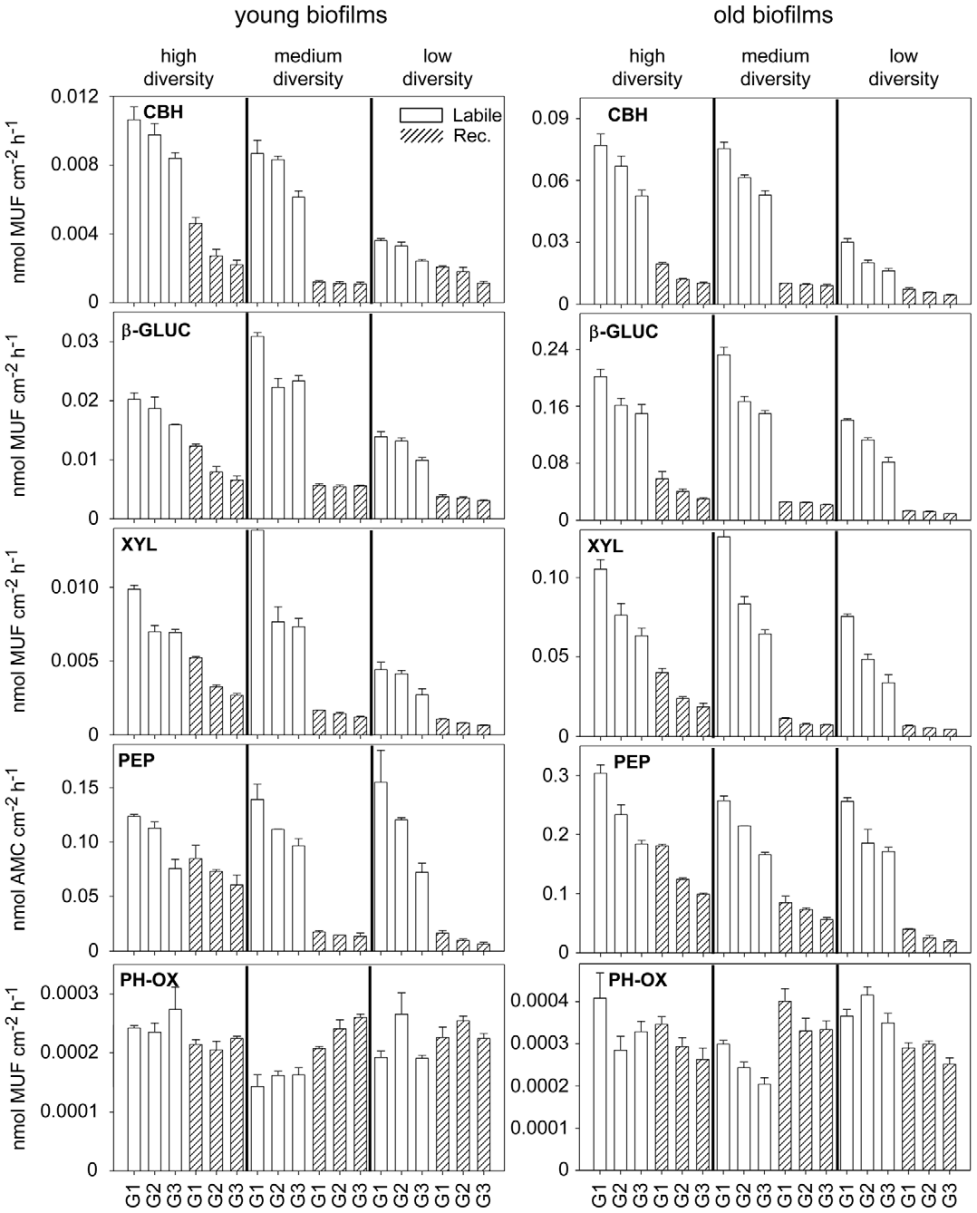
Overview of extracellular enzymatic activities in the different treatments at two sampling occasions (young and old biofilms, respectively). For each level of diversity the labile (white bars) and recalcitrant (hatched bars) treatments are for samples from the inflow (G1), mid section (G2) and outflow (G3) of each bioreactor. Mean ± s.e.m bars were shown, N = 3. Abbreviations: CBH: cellobiohydrolase, ß-GLUC: beta-glucosidase, XYL: xylosidase, PEP: leucine-aminopeptidase, PH-OX: phenoloxidase.

The absolute activities of the five enzymes were strongly affected by the age of the bioreactors, with old biofilms showing greater activities than young biofilms (MANOVA, P<0.001, Fig. 2). We could also observe pronounced differences among the carbon and diversity treatments and along the gradient from the in- to the outflow of the bioreactors (Fig. 2). For all enzymes, the activities were generally greater in the labile than in the recalcitrant carbon treatment, with the exception of phenoloxidase which was rather low and similar for each substrate. In the recalcitrant treatment, we generally found a decrease in extracellular enzyme activities from high to low diversity. This pattern differed in the labile treatment. For example, old biofilms with medium diversities achieved similar enzyme activities as the high diversity treatment (see Text S5 for a more detailed comparison of results obtained by the enzyme activity assays).

The change in the ratio between absorbance at 250 nm and DOC (Figure S1) during the 10 d periods of both test runs was positive in bioreactors fed with labile carbon. In bioreactors fed with recalcitrant carbon, this ratio remained unchanged in young biofilms and was negative in old ones. The diversity treatment did not influence these patterns of DOC utilization.

Reactor-wide oxygen consumption rates in the beginning of the test periods ranged between 0.10 and 0.50 mg O_2_h^−1^ in bioreactors fed with labile carbon and between 0.04 and 0.15 mg O_2_h^−1^ in bioreactors fed with recalcitrant carbon. Oxygen consumption rates dropped during the two test periods in the labile carbon treatment but remained similar in the recalcitrant carbon treatment (Figure S2). Diversity did not influence oxygen consumption in the labile (ANOVA _MS 0.07, d.f. 8_ P = 0.50) and the recalcitrant (ANOVA _MS 0.01, d.f. 8_ P = 0.31) treatment.

**Figure 3:**
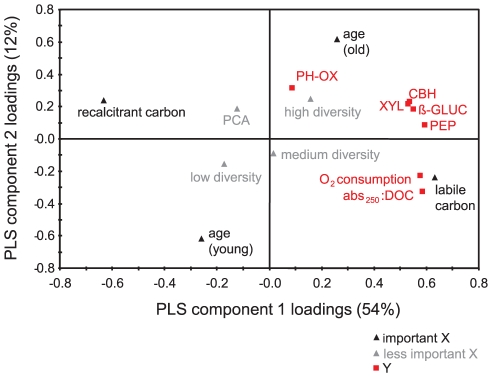
PLS loading plot of community functioning. The plot depicts the correlation structure between X and Y and can be interpreted by drawing a line from any Y-variable through the origin and by identifying the position of orthogonal projections of the X-variables. Factors (X) close to and on the same side of the origin (0,0) are positively related to the response (Y), and factors more distant from the origin are more important. Y-variables close to each other are correlated. Factors are separated according to their importance (VIP). Abbreviations: CBH: cellobiohydrolase, ß-GLUC: beta-glucosidase, XYL: xylosidase, PEP: leucine-aminopeptidase, PH-OX: phenoloxidase, abs250:DOC: change in the ratio of absorbance at 250 nm to dissolved organic carbon concentration over 10 days, PCA: scores on the first axis of Principal Component Analysis of the T-RFLP results.

Two significant components were identified using PLS (Fig. 3), which yielded a cumulative R^2^Y of 0.66 and a Q^2^Y of 0.60. Variable influence on projections (VIP) indicated that carbon source and age were overall important X variables (VIP≥1), while the diversity treatment and community composition (PCA scores) seem less important (VIP<0.5) in predicting Ys. Based on individual VIP, the presence of labile carbon was important for peptidase, glucosidase and xylosidase activity, as well as for oxygen consumption and the change in the abs_250_:DOC ratio. For cellobiohydrolase and phenoloxidase, however, age was relatively more important.

### Multifunctionality

**Figure 4:**
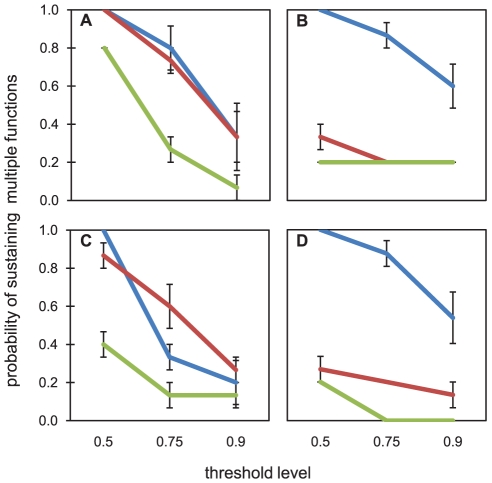
Probabilities for all 5 studied enzymes to be active above a threshold level of 0.5, 0.75 or 0.9 of the maximum activities. Shown are the probabilities for bioreactors fed with labile (A and C) and recalcitrant (B and D) organic carbon. Differences in the likelihood of sustaining joint functioning for young (A and B) and old (C and D) biofilms are shown. Blue lines represent the high diversity treatment, red lines the medium diversity treatment and the green lines the low diversity treatment. Error bars indicate s.e.m. N = 3.

The probability of finding all five enzymes active above a certain threshold was strongly affected by diversity (Fig. 4). In general, at higher diversities, the probability of retaining multifunctionality was much greater than to treatments where diversity was reduced. As expected, likelihoods decreased drastically when the threshold level was increased, however, the high diversity treatment performed above the 0.5 threshold in all scenarios. Bioreactors fed with the labile carbon source were less affected by the loss of diversity. In bioreactors exclusively fed with recalcitrant carbon, the effects of diversity on multifunctionality were very pronounced, with a reduction of the likelihood to sustain enzyme activities from 1 at highest diversity to 0.2 at lowest diversity (threshold level 0.5). Old biofilms had a reduced likelihood of all enzymes being active, especially when diversity was low.

## Discussion

An ongoing debate in ecology revolves around how bacterial diversity and ecosystem function are related. Here we present experimental estimates of the effects of loss of diversity on multifunctionality (Fig. 4). In natural biofilms, the interplay of several enzymes determines the processing of organic carbon. Insights into how the outcome of complex interactions of these enzymes and their substrates depends on diversity, requires a multifunctional perspective. Ecosystem functioning is the joint effects of multiple constituent functions and cannot be expressed as the average of individual functions, because a decline in one function cannot be compensated by an increase in another function [12]. These relationships can be assessed by defining a specific threshold for each function, and when a function drops below this threshold, overall ecosystem functioning is no longer sustained [13]. Moreover, there might be metabolic and/or stoichiometric constraints on the synthesis of extracellular enzymes [31]. A threshold relative to the maximal enzyme activity reduces the risk of a bias introduced by such constraints. We chose to investigate a range of thresholds, from values where the high diversity treatment sustained multifunctionality (0.5) to more stringent conditions (0.9). The selection of thresholds was based on Gamfeldt et al. [12] and the results of a recent meta-analysis [4] which reports an average diversity effect size corresponding to a 40% reduction in functioning. The likelihoods of sustaining multifunctionality converged under stringent conditions (threshold: 0.9), which could reflect the importance of individual species performing extraordinarily well [4].

When functional redundancy among species occurs, single ecosystem functions tend to saturate at relatively low levels of diversity [5]. However, more species are needed to sustain a higher number of functions, i.e. redundancy decreases when more functions are considered. In an analysis of published data, Gamfeldt and co-authors [12] found that, compared to plant communities, bacterial communities feature a high degree of multifunctional redundancy. However, these results were derived from biomass, consumer biomass and decomposition measurements. In the present study, multifunctionality of bacterial communities with respect to the activity of the studied extracellular enzymes was lost with decreasing diversity to an extent that implies a relatively low level of multifunctional redundancy. Multifunctional redundancy is conceptually based on the idea that trade-offs exist for species performing several functions [8], [12]. Our results could reflect that functional trade-offs might be stronger between more specialized functions than in functions like biomass or respiration. Our observation is in line with the study of He et al. [14] who found a strong coupling of microbial diversity and multifunctionality in soils. They used backward statistical selection to extract OTUs with a positive effect on specific functions and estimated the effects of diversity on multiple functions. The statistical selection procedure does not account for compensatory growth or physiological adaptations, which might be of pivotal importance to natural communities which encounter a loss of diversity. Our experimental approach corroborates the findings of He et al. [14], while at the same time taking into account the possibility that fast growth rates and physiological versatility of the bacterial community might have enabled compensation for the loss of functions along with the loss of certain taxa. This is reflected in our finding that substrate composition influences the way multifunctionality is maintained (Fig. 4). Multifunctionality was less impaired by reduced diversity in the labile medium compared to the recalcitrant medium, where only the highest diversity was likely to sustain multiple functions at the same time. This reflects the ability of bacterial species to adjust to their environment, which consequently affects multifunctional redundancy.

We found that bacterial biofilms differ in their composition and function on short temporal and small spatial scales. Multifunctionality was less affected in young compared to old biofilms, which might reflect that communities in later successional stages may establish more complex structures [17]. Biodiversity effects on functioning are known to become stronger over time [8], [32] and old biofilms may be richer in facilitative interactions among species. Also, with increasing duration more biomass might accumulate in the biofilms and affect functions like respiration rate. However, we found that abundance on the beads rather decreased during the experiment (Text S6).

The dilution of a complex community to create a gradient in diversity, as applied in this study, is successful in generating significant effects on functioning [24]. The molecular fingerprinting technique (T-RFLP), however, is restricted in the ability to reveal such gradients since it detects only the most abundant members of the community [33], [34]. The dilution-to-extinction approach results in non-stochastic removal of rare organisms [34] and the presence of a species at a certain dilution step depends on its initial relative abundance. A rare species is more likely to be removed by dilution first, while a dominant member of the community remains also in the more diluted cultures. Support for the establishment of differences in diversity might be derived from non-metric multidimensional scaling, which showed that similarities between treatments decreased with increasing dilution (Fig. 1A). The high diversity treatments were rather similar while the medium and low diverse treatments were more dissimilar. A reason for this could be that the likelihood of the community including a specific set of species was high at high diversities, allowing more similar communities to develop from a rich inoculum. When diversity in the inoculum is reduced, however, the likelihood that it contains a certain set of species decreases, which ultimately leads to more dissimilar communities. Using a dilution-to extinction approach allows including a large number of different species, derived from a naturally occurring community and maintaining the evenness structure along the diversity gradient. Alternative approaches require combinatorial experimental designs [35] which are most commonly achieved by artificial assembly of communities [36], [37]. These experiments allow controlling for community composition; however they are restricted in their complexity and criticized for the limited level of diversity that can be manipulated [8]. Moreover, a rare species is more likely to be removed by dilution, while a dominant member of the community most probably remains also in the most diluted cultures. In natural ecosystems, species respond differently to environmental fluctuations and rare species are more prone to extinction. Therefore, elimination of species from natural ecosystems is most likely non-stochastic [38] and the loss of taxa according to patterns of dominance and rarity resulting from dilution-to-extinction might be a realistic model of natural extinction patterns.

Most research on the relationship between diversity and functioning addresses rather uniform environments [8] and only recently has the influence of resource complexity on microbial diversity been brought into focus [39], [40]. Species-specific trade-offs in the ability to exploit resources might enhance complementarity among species and one could expect that functioning would increase with increasing biodiversity as well as with increasing resource complexity [39]. Although the diversity treatment had a significant impact on functioning, effects of diversity and community composition were subordinate to the environmental and successional factors (carbon source composition and age, respectively) as shown by the PLS model (Fig. 3). Accordingly, Singer et al. [40], provided evidence for the effects of flow heterogeneity on microbial diversity and functioning. Biofilms in different stream sections differed in their abilities to utilize labile (glucose) and recalcitrant sources of organic carbon, depending on environmental heterogeneity. Our results extend this conclusion to more naturally occurring labile compounds such as cellobiose and the dipeptide leucine-proline.

The loss of activity along the diversity gradient was not equal for all enzymes. In the presence of labile substrate, the activities of enzymes related to degradation of complex polysaccharides such as cellobiohydrolase showed a greater reduction than β-glucosidase, β-xylosidase and leucine-aminopeptidase. Leucine-aminopeptidase and β-glucosidase are involved in the decomposition of simple polysaccharides and peptides into energetically favorable bacterial substrates (glucose and leucine, respectively) (Table S1). Leucine-aminopeptidase and β-glucosidase are considered to be commonly expressed by a large fraction of the bacterial community. Cellobiohydrolase and phenoloxidase might be less common and not expressed by all bacterial species [41]. However, the latter might also be more plastic in their expression, only being expressed when labile compounds are no longer available and communities forced to degrade complex substances. Such plasticity can result in patterns similar to functional redundancy [42], where several species perform the same functions and decreasing diversity can be compensated through compositional adjustments [43]. Using PLS modeling, we show that bacterial community composition (PCA scores) was relatively unimportant for the observed patterns in functioning, which rather supports the importance of plasticity.

This study demonstrates that reduced diversity impairs multifunctionality to a much larger extent than it diminishes individual functions. Hence, bacterial communities may be considerably less redundant than previously suggested [44] based on experiments and observations focusing on specific, individual functions.

## Supporting Information

Figure S1 **Changes in ratio of absorbance at 250 nm to DOC concentration between the start (T0) and end (T10) of the two tests (young and old biofilms) for bioreactors fed with labile and recalcitrant carbon, respectively.** Positive values indicate an increase in the fraction of humic compounds relative to the total organic carbon pool. Error bars indicate ± s.e.m, N = 9.
(EPS)

Figure S2 **Differences in oxygen concentration in the in- and outflow of the bioreactors.** Results are shown for the two test periods, young biofilms (A and B) and old biofilms (C and D). Initial oxygen consumption was greater in bioreactors fed the labile (A and C) compared to bioreactors fed the recalcitrant carbon source (B and D). There were no differences between the high (◊), medium (○) and low (Δ) diversity treatments.
(EPS)

Table S1 **Overview of extracellular enzyme activities measured along gradients within the bioreactors.** The specific reaction performed and EC (Enzyme Commission) numbers are shown for each enzyme. The substrate analogues used for the enzyme activity measurements are also indicated (MUF: methylumbellyferone, AMC: aminomethylcoumarin).
(DOCX)

Text S1 **DNA extraction and terminal restriction fragment length polymorphism (T-RFLP) analysis.**
(DOCX)

Text S2 **Flow Cytometry.**
(DOCX)

Text S3 **Extracellular enzyme activities.**
(DOCX)

Text S4 **PLS model evaluation.**
(DOCX)

Text S5 **Extracellular enzyme activity comparisons.**
(DOCX)

Text S6 **Abundance.**
(DOCX)
