# Supplementary material for: Multifunctionality and Diversity in Bacterial Biofilms
Source: PLoS One. 2011 Aug 5;6(8):e23225. doi: 10.1371/journal.pone.0023225 (PMC3151291; doi:10.1371/journal.pone.0023225)
Supplement: Table S1 — Overview of extracellular enzyme activities measured along gradients within the bioreactors. The specific reaction performed and EC (Enzyme Commission) numbers are shown for each enzyme. The substrate analogues used for the enzyme activity measurements are also indicated (MUF: methylumbellyferone, AMC: aminomethylcoumarin). (DOCX) [file pone.0023225.s003.docx]

| **enzyme** | **EC** | **reaction** | **substrate**  **analogue** | **substrate source in environment** | **reference** |
| --- | --- | --- | --- | --- | --- |
| cellobiohydrolase | 3.2.1.91 | cellulose -> cellobiose | 4-MUF- β-D- cellobioside | plant cell walls | [1] |
| β-glucosidase | 3.2.1.21 | cellobiose -> glucose | 4-MUF-β-D-glucopyranoside | plant cell walls, decaying organisms, algal and bacterial exudates | [1] |
| β-xylosidase | 3.2.1.37 | xylobiose-> xylose | 4-MUF-7-β-D-xyloside | plant cell walls | [2] |
| leucine-aminopeptidase | 3.4.11.1 | peptide -> leucine | L-leucine-4-methyl-7-coumarinylamide | decaying organisms, algal and bacterial exudates | [3] |
| phenoloxidase | 1.14.18.1 | oxidation of phenolic compounds (i.e. lignin) | L-3,4 dihydroxy-phenylalanine | plant cell walls | [4] |

**References**

1. Deshpande V, Eriksson KE (1988) 1,4-Beta-Glucosidases of Sporotrichum-Pulverulentum. Methods in Enzymology 160: 415-424.

2. Lachke AH (1988) 1,4-Beta-D-Xylan Xylohydrolase of Sclerotium-Rolfsii. Methods in Enzymology 160: 679-684.

3. Hoppe H-G, Kim S-J, Gocke K (1988) Microbial Decomposition in Aquatic Environments: Combined Process of Extracellular Enzyme Activity and Substrate Uptake. Applied and Environmental Microbiology 54: 784-790.

4. Sinsabaugh RL, Osgood MP, Findlay S (1994) Enzymatic Models for Estimating Decomposition Rates of Particulate Detritus. Journal of the North American Benthological Society 13: 160-169.
