# Supplementary material for: Multifunctionality and Diversity in Bacterial Biofilms
Source: PLoS One. 2011 Aug 5;6(8):e23225. doi: 10.1371/journal.pone.0023225 (PMC3151291; doi:10.1371/journal.pone.0023225)
Supplement: Text S1 — DNA extraction and terminal restriction fragment length polymorphism (T-RFLP) analysis. (DOCX) [file pone.0023225.s004.docx]

*Supporting Text S1 DNA extraction and terminal restriction fragment length polymorphism (T-RFLP) analysis*

DNA extracts were used as templates for PCR amplification of the 16S rRNA genes with the universal primers 27-forward, labelled with hexachlorofluorescein (HEX) and unlabelled 519-reverse. Thermocycling was carried out with a MyGene MG 96 Thermocycler (Longene Scientific Instruments, Hangzhou, China) using an initial 30 s denaturation at 98ºC, 28 cycles of 98ºC for 10 s, 50ºC for 30 s and 72ºC for 30 s followed by a final 7 min extension step at 72ºC. Pooled PCR products were purified using MultiScreen PCRµ96 plates (Millipore, Billerica, MA, USA).
