# Supplementary material for: Multifunctionality and Diversity in Bacterial Biofilms
Source: PLoS One. 2011 Aug 5;6(8):e23225. doi: 10.1371/journal.pone.0023225 (PMC3151291; doi:10.1371/journal.pone.0023225)
Supplement: Text S2 — Flow Cytometry. (DOCX) [file pone.0023225.s005.docx]

*Supporting Text S2 Flow Cytometry*

The samples were analyzed with a Cyflow Space (Partec, Görlitz, Germany) flow cytometer equipped with a 96 well-plate autosampler. For this purpose, 200 µL subsamples were loaded into a 96 well-plate and stained for 10 minutes with a 1.25 µM Syto13 solution. The detector settings were optimized for the samples to 465 FS1 (fluorescence at 508 nm) and 245 FFC (forward scatter).
