# Supplementary material for: Multifunctionality and Diversity in Bacterial Biofilms
Source: PLoS One. 2011 Aug 5;6(8):e23225. doi: 10.1371/journal.pone.0023225 (PMC3151291; doi:10.1371/journal.pone.0023225)
Supplement: Text S3 — Extracellular enzyme activities. (DOCX) [file pone.0023225.s006.docx]

*Supporting Text S3 Extracellular enzyme activities*

A pre-study with two extra bioreactors was performed in order to determine the saturation curves for each of the five enzymes. Based on these results, we used 0.3 mmol/L substrate for β-glucosidase, β-xylosidase and leucine-aminopeptidase, 0.8 mmol/L substrate for cellobiohydrolase and 5 mmol/L for the measurement of phenoloxidase activity, to achieve V_max_ estimates. For the measurements, substrates were added to 4 mL of beads covered with 4 ml of artificial lake water medium (carbon free). The samples were kept for 2 h in the dark, at 20ºC and under continuous shaking. Cellobiohydrolase, however, was incubated for 5 h. Blanks and standards of MUF and AMC were included. Incubations were terminated by adding glycine buffer (pH 10.4, 1/1 vol/vol), and fluorescence was measured at 360/465nm excitation/emission for MUF and AMC using a plate reader (Ultra 384, Tecan, Switzerland).
