# Supplementary material for: Multifunctionality and Diversity in Bacterial Biofilms
Source: PLoS One. 2011 Aug 5;6(8):e23225. doi: 10.1371/journal.pone.0023225 (PMC3151291; doi:10.1371/journal.pone.0023225)
Supplement: Text S4 — PLS model evaluation. (DOCX) [file pone.0023225.s007.docx]

*Supporting Text S4 PLS model evaluation*

Most variables could be well modeled, as indicated by R^2^Ys ranging between 0.66 and 0.90 and Q^2^Ys between 0.59 and 0.87. Phenoloxidase, however, obtained a R^2^ value of 0.23 and a Q^2^ of 0.10. Although phenoloxidase was measured close to the detection limit and lowers the overall quality of the PLS model, we decided to keep it in the analysis. The PLS model was validated by comparing the goodness of fit of the model with the goodness of fit with models (100 permutations), using randomized Y-variables. Model validation showed that the amount of explained variance (R^2^Y) and predictive power (Q^2^Y) that resulted due to chance was below 4% for all Y-variables, which indicates that the original PLS model was valid.
