# Supplementary material for: Multifunctionality and Diversity in Bacterial Biofilms
Source: PLoS One. 2011 Aug 5;6(8):e23225. doi: 10.1371/journal.pone.0023225 (PMC3151291; doi:10.1371/journal.pone.0023225)
Supplement: Text S5 — Extracellular enzyme activity comparisons. (DOCX) [file pone.0023225.s008.docx]

*Supporting Text S5 Extracellular enzyme activity comparisons*

Communities with low diversity expressed ca. 65% of the β-glucosidase and β-xylosidase activity of the high diversity treatments. Activity of cellobiohydrolase in low diversity treatments was one third of that in the high diversity treatments, both for young and old biofilms. In contrast, the smaller peptidase activity in treatment with less bacterial diversity was much less evident. This reduction was not significant for the young biofilms, and for the old biofilms 88% and 84% of the maximal activity occurred in the medium and low diversity treatments, respectively.

Except of phenoloxidase, enzyme activities were generally higher close to the inflow than in the mid-section or the outflow. This could reflect the decreasing availability of substrates along the flow path in the bioreactors
