# Supplementary material for: Multifunctionality and Diversity in Bacterial Biofilms
Source: PLoS One. 2011 Aug 5;6(8):e23225. doi: 10.1371/journal.pone.0023225 (PMC3151291; doi:10.1371/journal.pone.0023225)
Supplement: Text S6 — Abundance. (DOCX) [file pone.0023225.s009.docx]

*Supporting Text S6 Abundance*

The number of cells on the beads was highest in the medium diversity treatment (median: 2.01×10^7^ cells mL^-1^ beads), followed by the high diversity treatment (median: 1.14×10^7^ cells mL^-1^ beads) and the low diversity treatment (1.12×10^7^ cells mL^-1^ beads) (ANOVA,_MS 4.3x10_^13^_, d.f. 107_, P = 0.02). Abundance on the beads was higher in the recalcitrant (median: 2.23×10^7^ cells mL^-1^ beads) compared to the labile treatments (3.67×10^6^ cells mL^-1^ beads) (Student’s ttest P < 0.01). Moreover, there was a decrease in the number of cells on the beads during the experiment (young biofilms: median: 2.25×10^7^ cells mL^-1^ beads; old biofilms: median: 1.21×10^7^ cells mL^-1^ beads, Student’s ttest P = 0.02).
